# Protective and therapeutic efficacy of phytogenic botanical blend and multi-strain probiotic in Avian Pathogenic *Escherichia coli*-challenged broilers

**DOI:** 10.5713/ab.260539

**Published:** 2026-09-01

**Authors:** Antara Anup Guha, Pratik Hivraj Barsinge, Bhagyalaxmi Chinnavadu Junnuru, Amit Chandrakant Kini

**Affiliations:** 1 Department of Research and Development, Nutranovel Additives Pvt. Ltd., Bhiwandi, India

**Keywords:** Avian Pathogenic *Escherichia coli*, Broilers, Growth Performance, Hematology, Phytogenic Botanical Blend, Prophylactic

## Abstract

**Objective:**

Avian pathogenic *Escherichia coli* (APEC)-induced colibacillosis poses a significant challenge in broiler production, necessitating alternatives to antibiotics due to emergence of antimicrobial resistance. However, limited information is available regarding the efficacy of phytogenic botanical blend (PBB). Therefore, the study was conducted to evaluate the effectiveness of a PBB, either alone or in combination with a liquid formulation, in broiler chickens under an APEC challenge, in comparison with a multi-strain probiotic (MSP).

**Methods:**

A total of 384 one-day-old male “Vencobb 430Y” broiler chicks were randomly allocated to eight treatments with four replicates of 12 birds each for 42 days. Treatments included negative and positive controls, PBB supplementation (with or without APEC challenge), PBB combined with liquid supplementation under prophylactic and therapeutic regimens, and MSP-based programs. The APEC challenge was initiated at 10 days of age (1×10^8^ CFU/bird). Growth performance parameters, mortality rates, and European production efficiency factor were recorded. On day 14 (4 days post-infection), samples were collected for hematological, serum biochemical, and *E. coli* enumeration analyses, along with lesion scoring of visceral organs.

**Results:**

Growth performance parameters were not significantly affected (p>0.05) by either PBB or MSP supplementation in chickens with APEC challenge. Survival rates enhanced with PBB, exhibiting superior outcomes in prophylactic applications compared to therapeutic ones. Hematology remained unchanged, while serum biochemical markers (aspartate aminotransferase, Alkaline phosphatase, bilirubin, total protein) were significantly altered, indicating liver involvement, with reduced stress under prophylactic PBB. Birds in the PC+PBB (Ther) group showed 0 for abdominal and thoracic lesion score. *E. coli* counts in fecal content at day 16 were significantly reduced (p<0.05) in the groups PC+PBB, PC+MSP and PC+PBB (Ther) compared to PC.

**Conclusion:**

Overall, the powder based PBB, particularly when used for prevention, showed promising potential to mitigate the adverse effects of APEC challenge. However, as most parameters did not reach statistical significance, these findings should be considered supportive rather than definitive.

## INTRODUCTION

Avian pathogenic *Escherichia coli* (APEC), an extra-intestinal pathogenic strain, causes significant infections in poultry including chickens, turkeys, and ducks, spreading via contaminated feed, water, and fecal-oral or aerosol routes [1]. It leads to diseases such as perihepatitis, air sacculitis, pericarditis, and cellulitis, resulting in high mortality (up to 20%), morbidity, and reduced production. APEC imposes major economic losses globally, with an estimated $40 million annually in the U.S. due to carcass condemnation, and mortality in young birds reaching up to 53.5% [2]. Its pathogenicity is driven by key virulence factors such as fimC, papC, and iucD, which enable adhesion, iron acquisition, immune evasion, and systemic spread [3].

The emergence of multidrug-resistant APEC, with carbapenems remaining among the few effective antibiotics, has intensified concerns in poultry production [4]. Global restrictions on antibiotic growth promoters have accelerated the search for alternatives [5]. Probiotics (e.g., *Bacillus*, *Saccharomyces*) and phytogenic feed additives such as chicory (*Cichorium intybus*), rosemary (*Rosmarinus officinalis*), and grape seed (*Vitis vinifera*) show promise due to their prebiotic, antimicrobial, antioxidant, and gut health-enhancing properties [6]. Chicory improves gut health via inulin-type fructans [7], rosemary enhances immunity and microbial balance through phenolics and flavonoids [8], and grape seed supports intestinal health through polyphenols [9]. These additives also improve growth performance, hamatology and biochemical parameters in poultry [10].

Although phytogenic compounds and botanical mixtures have previously been investigated in poultry challenged with APEC [11], limited information is available regarding the efficacy of a specific multi-component phytogenic botanical blend (PBB) in APEC-challenged broilers, particularly under prophylactic and therapeutic application strategies.

Therefore, this study aims to evaluate the relative efficacy of selected probiotics and PBB in broilers under APEC challenge, focusing on growth performance, hematological and biochemical parameters, lesion scoring, and fecal and cecal *E. coli* load.

## MATERIALS AND METHODS

### Animal care

The study was conducted at the research facility of Agrivet Research and Advisory Pvt. Ltd., located at Village: Jhampa, P.O. Kaliani, P.S.: Deganga, North 24 Parganas, PIN 743423.

### Phytogenic botanical blend and multi-strain probiotic

The present study was designed to evaluate the synergistic effects of selected phytogenic extracts at defined concentrations. A standardized phytogenic formulation comprising *R. officinalis*, *C. intybus*, and *V. vinifera* was prepared for administration via both feed and drinking water and was designated as PBB. The raw materials were initially sieved using a vibratory separator to remove extraneous matter, followed by sequential blending and homogenization to ensure uniform distribution. The resulting mixture was subsequently incorporated into the basal diet.

The feed-supplemented multi-strain probiotic (MSP) consisted of a consortium of beneficial microbial strains, including *Bacillus megaterium, Bacillus subtilis, Bacillus coagulans, Bacillus mesentericus, Bacillus polymyxa* and *Bacillus licheniformis*, supplemented with *Saccharomyces boulardii*. In addition, a separate MSP formulation was administered via drinking water, comprising *Bacillus subtilis* as the active microbial component along with maltodextrin and sodium bicarbonate as carriers.

### Preparation of phytogenic botanical blend

Selected plant materials such as bark and leaves were collected, thoroughly washed, and dried at 50°C for 2–3 days. The dried materials were finely ground and extracted individually with sterile distilled water. The extracts were subjected to sequential centrifugation to remove particulate matter, followed by filtration through 0.65, 0.45, and 0.22 μm membrane filters to obtain clarified extracts. The filtered extracts were combined in predetermined proportions to prepare the PBB and stored in sterile containers at 4°C in the dark until further use. The final PBB was prepared to contain not less than 50 mg/ 100 g total flavonoids, 300 mg/kg total alkaloids, 800 mg/100 g tannins, and 7,000 mg/kg total polyphenols [12].

### Birds’ management and experimental design

A total of 384 one-day-old male broiler chicks (Vencobb 430Y) were procured and reared on wood-shaving litter in floor pens. Chicks were individually weighed and randomly allocated to eight treatment groups (randomized block design), each with four replicates of 12 birds. The eight experimental groups were defined as follows: Negative control without APEC challenge (NC), positive control with APEC challenge (PC), NC supplemented with PBB (NC+PBB), PC supplemented with PBB (PC+PBB), PC supplemented with PBB following prophylactic regimen (PC+PBB Prophy), PC supplemented with PBB under therapeutic condition (PC+PBB Ther), PC supplemented with MSP (PC+MSP) and PC supplemented with MSP following therapeutic regime (PC+MSP Ther). The prophylactic regimen was administered for 3 days prior to and 7 days following the APEC challenge, whereas the therapeutic regimen was administered for 10 consecutive days after the onset of clinical signs, including reduced activity (lassitude), drooping wings, and somnolence, as previously described [13]. The regimens were adapted from a previously reported broiler challenge study to evaluate preventive and therapeutic efficacy in the present APEC model [14]. The detailed treatment regime is described in Table 1.

The 42-day trial included three-phase corn–soybean diets (starter 0–10 d, grower 11–24 d, finisher 25–42 d). Diets were pelleted at 80±2°C, 2.5 kg/cm^2^ for 35–40 s. Drinking water was non-sanitized, and feed and water were provided ad libitum using manual feeders and drinkers. Table 2 presents the composition and calculated nutrient composition of the basal diet.

All birds were vaccinated against infectious bronchitis (day 0), Newcastle disease (days 6 and 20), and infectious bursal disease (day 12). Mortality and body weight were recorded throughout the trial, and post-mortem examinations were performed to determine the cause of death. Strict biosecurity measures, including litter guards, separate equipment, and dedicated personnel, were maintained to prevent cross-contamination.

### Growth performance parameters

Live body weight (LBW), body weight gain (BWG), feed intake (FI), and feed conversion ratio (FCR) were recorded on days 10, 25, 35, and 42 of the experiment. FCR was calculated by the formula given below. Daily mortality was monitored throughout the study period, and overall liveability was calculated. Necropsy examinations were performed to determine the probable cause of mortality. At the end of the experimental period, the European production efficiency factor (EPEF) was calculated using standard methodology based on liveability, body weight, age, and FCR. Feed cost was also evaluated at the end of the experimental period.


(1) $$\text{FCR}=\frac{Feed\;consumption\;up\;to\;the\;respective\;timepoints}{Body\;weight\;gain\;up\;to\;the\;respective\;timepoints}$$

(2) $$\text{EPEF}=\frac{Liveability\;(\%)\times LBW\;(Kg)\;at\;42nd\;day\times 100}{FCR\times Age\;at\;marketing\;(42\;d)}$$

### Avian pathogenic *Escherichia coli* challenge

The challenge organism was a field isolate of *E. coli* recovered from poultry farm litter collected from a commercial broiler farm in West Bengal, India. Species identity was confirmed by PCR targeting the 16S rRNA gene, and pathogenic characterization was performed by PCR detection of the APEC-associated virulence genes fimC, papC, and iucD. The presence of these adhesion- and iron acquisition-related virulence determinants supported the classification of the isolate as an APEC strain suitable for experimental challenge.

### Induction of colisepticemia

A standardized APEC infection challenge model from Agrivet Research and Advisory, molecularly characterized (fimC, papC, iucD) and validated to induce colisepticemia, was used. A working culture was grown in nutrient broth at 37°C for 18–24 h to prepare ~1×10^8^ CFU/mL inoculum. At 10 days of age, birds in challenged groups received 1×10^8^ CFU/bird (1 mL) via oral gavage, while controls received sterile saline.

### Euthanasia of birds

On day 14 of the trial (4-day post-infection) one bird from each replicate in each treatment group (n = 32 birds) with body weight closest to the respective pen was selected for detailed sampling. Selected birds were humanely euthanised by mechanical stunning followed immediately by exsanguination. Exsanguination was performed by severing the jugular vein and carotid artery, followed by transection of the trachea using a sterile knife.

### Lesion scoring of visceral organs

On day 14, one bird per replicate from each treatment (n = 32) was euthanized and examined for gross lesions, as previously described [13]. The personnel involved in lesion scoring were blinded to the treatment allocation. Throughout the study, treatment identities were concealed from investigators performing necropsy and lesion assessment to minimize observer bias. Liver was assessed for colisepticemia, and serosal surfaces for perihepatitis, pericarditis, and air sacculitis based on macroscopic APEC criteria. Lesions were graded as mild, moderate, or severe [15]. Individual organ scores were recorded, with higher scores indicating greater severity of colibacillosis.

### Bacteriological analysis of fecal and cecal content

After euthanasia, the posterior cecum was ligated, aseptically excised, and transported in sterile bags. About 1 g of cecal content was homogenized in sterile phosphate-buffered saline, serially diluted, and plated (100 μL, duplicate) on Eosin Methylene Blue agar (Hi-Media M317), incubated at 37°C for 24–36 h; metallic green colonies were counted as *E. coli* and expressed as log_10_ CFU/g. Fecal samples were collected from each pen on day 9 (pre-challenge) and day 16 (6 DPI), pooled per pen, processed similarly, and mean duplicate counts were used to calculate CFU/g.

### Blood collection and serum biochemistry

On day 14 (4 DPI), four birds per treatment (n = 32) were randomly sampled, and approximately 5 mL of blood was aseptically collected from the left brachial vein using sterile needles and vacutainers. About 2.5 mL was placed in plain tubes for serum, and the remaining in 4% EDTA tubes for hematology. Serum was obtained after clotting and centrifugation (800×g, 10 min), stored at −20°C, and analysed photometrically for total protein (TP), total bilirubin, aspartate aminotransferase test (AST), alanine aminotransferase test (ALT), Alkaline phosphatase test (ALP) using a semi-automated analyser (Nanolab 200; Trivitron Healthcare) with commercial kits. EDTA blood was used for complete blood count and differential leukocyte count.

### Statistical analysis

Data are expressed as mean±standard error (SE). Group differences were assessed by one-way ANOVA [16], followed by Tukey’s post hoc test [17]. Mortality data were analysed using the Kruskal–Wallis test [18], followed by Dunn’s multiple-comparison test [19] for pairwise comparisons. A p-value of less than 0.05 was considered statistically significant. Statistical analyses were carried out using GraphPad Prism (ver. 10.3.0).

## RESULTS

### Live body weight

Table 3 presents LBW of broiler chickens across the different experimental groups. No significant differences (p>0.05) in LBW were observed among the treatment groups on days 10, 24, 35, or 42. Although minor numerical variations were observed, the supplemented groups generally exhibited higher LBW than their respective control groups; however, these differences were not statistically significant. On day 42, the NC+PBB group exhibited the highest numerical LBW, followed by the PC+PBB, PC+MSP, PC+PBB (Prophy), PC+MSP (Ther), PC+PBB (Ther), NC, and PC groups.

### Body weight gain

Table 4 presents BWG of broiler chickens across the different experimental groups. No significant differences (p>0.05) in BWG were observed among the experimental groups during any growth phase or over the entire experimental period (0–42 d). Although numerical variations were observed, the supplemented groups generally exhibited higher BWG than their respective control groups. Over the entire experimental period, the NC+PBB group recorded the highest numerical BWG among the non-challenged groups, whereas the PC+PBB and PC+MSP groups recorded the highest numerical BWG among the challenged groups, followed by the PC+PBB (Prophy) group. However, these differences were not statistically significant (p>0.05).

### Feed intake

No significant differences (p>0.05) were observed in FI among the experimental groups during the starter, grower, finisher, or cumulative phases (Table 5). Minor numerical variations were observed. Although the PC+PBB and PC+MSP (Ther) groups showed numerically higher cumulative FI (0–42 d), these differences were not statistically significant (p>0.05).

### Feed conversion ratio

No significant differences (p>0.05) in FCR were observed among the experimental groups during the starter, grower, finisher, or cumulative phases (Table 6). Although minor numerical variations were observed, the supplemented groups generally exhibited lower FCR than their respective control groups. Over the entire experimental period (0–42 d), the NC+PBB group recorded the lowest numerical FCR among the non-challenged groups, whereas the PC+PBB (Prophy) group recorded the lowest numerical FCR among the challenged groups, followed by the PC+MSP and PC+PBB groups. However, these numerical differences were not statistically significant.

### Average mortality percentage

#### Without challenged conditions

The NC and NC+PBB groups recorded no mortality during the starter (0 to 10 days), grower (11 to 24 days), and finisher phase I (25 to 35 days) periods. In contrast, a comparable mortality rate was observed in both groups during finisher phase II (36 to 42), leading to similar cumulative mortality over the entire 0–42-day experimental period (Table 7).

#### With challenged conditions

No mortality was recorded in any experimental group during the starter phase (0–10 d). During the grower phase (11–24 d), mortality was reduced by 28.53%, 71.40%, 28.53%, and 42.87% in the PC+PBB, PC+PBB (Prophy), PC+MSP, and PC+MSP (Ther) groups, respectively, compared with the PC group, while the PC and PC+PBB (Ther) groups showed the highest mortality. Over the entire experimental period (0–42 d), mortality was reduced by 25.02%, 75.00%, 12.54%, and 25.02% in the PC+PBB, PC+PBB (Prophy), PC+MSP, and PC+MSP (Ther) groups, respectively, compared with the PC group. The overall Kruskal–Wallis test was significant (p = 0.0166), but no individual pairwise comparison remained significant after Dunn’s multiple-comparison adjustment.

### European production efficiency factor

Statistical analysis showed no significant differences (p>0.05) among the experimental groups (Table 7). The NC+PBB group recorded the highest numerical EPEF among the non-challenged groups, whereas the PC+PBB (Prophy) group recorded the highest numerical EPEF among the challenged groups, followed by the PC+PBB, PC+MSP, and PC+MSP (Ther) groups. However, these numerical differences were not statistically significant (p>0.05).

### Enumeration of *Escherichia coli* in cecal and fecal content

At 14 days (4 DPI), cecal *E. coli* counts showed a trend toward significance (p = 0.055), with the highest counts in PC and PC+MSP (Ther) and the lowest in PC+PBB (Table 8). Pre-challenge fecal counts at day 9 also tended toward significance (p = 0.053), with the highest values in PC+MSP, NC, PC, and NC+PBB and the lowest in PC+MSP (Ther). At 16 days (6 DPI), fecal *E. coli* counts were significantly reduced in PC+PBB, PC+MSP, and PC+PBB (Ther) compared with PC (p<0.05). Overall, PBB-supplemented challenged groups showed relatively lower *E. coli* counts, suggesting early modulation of gut microbiota.

### Lesion scoring of visceral organs

Tracheal lesions were minimal across all groups, with most birds exhibiting normal morphology (Score 0); only the NC and PC+PBB groups showed mild lesions (25%), while all other treatments remained 100% normal (Table 9). In contrast, abdominal air sac lesions varied among treatments, with the PC group showing greater lesion severity (50% normal, 25% mild, 25% moderate), confirming the impact of APEC challenge, whereas the NC group remained largely normal (75%). PBB supplementation exerted a protective effect, with the NC+PBB and PC+PBB (Ther) groups showing 100% normal conditions, while the PC+PBB and PC+PBB (Prophy) groups showed partial improvement (50% normal, 50% mild). MSP treatments demonstrated moderate efficacy, with 75% and 50% normal birds in the PC+MSP and PC+MSP (Ther) groups, respectively.

### Hematological and serum biochemical parameters

The effects of dietary treatments on hematological and serum biochemical parameters under APEC challenge are presented in Table 10. Most hematological indices, including TEC, TLC, heterophils, lymphocytes, H: L ratio, eosinophils, and basophils, were not significantly affected (p>0.05). The PC group showed increased TEC and reduced TLC, while monocyte percentage was significantly higher (p = 0.004) in the PC+PBB (Prophy) and PC+MSP groups. Among biochemical parameters, SGOT was significantly elevated (p<0.0001) in the PC group, indicating hepatic stress, whereas PBB alone maintained normal levels; higher SGOT values were observed in therapeutic and MSP-treated groups. ALP (p<0.0001), total bilirubin (p = 0.011), and TP (p = 0.006) were significantly affected, while SGPT (p = 0.071) and albumin remained unchanged. Overall, APEC challenge induced moderate hematological alterations and significant hepatic stress, whereas PBB, particularly under prophylactic supplementation, exhibited better protective effects than therapeutic interventions.

## DISCUSSION

The present study aimed to comparatively evaluate the prophylactic use of PBB and the therapeutic use of PBB and MSP administered through feed and drinking water in APEC-challenged broiler models. The ban on antibiotic growth promoters in Europe, along with increasing consumer awareness, has promoted the use of natural and safe feed additives such as herbal plant-based products, which are known to enhance appetite, improve digestion and physiological functions, and exert antioxidant effects in poultry [20].

At the end of the trial, PBB and MSP supplementation showed similar response and had no significant effect (p> 0.05) on LBW, BWG, FI and FCR in APEC-challenged birds. Similar results were reported by Aziza et al. [21], who observed no significant effects of rosemary oil supplementation on LBW, BWG, or FCR in *E. coli*-challenged quails. Conversely, Zhao et al. [22] reported improved (p<0.05) LBW and BWG with *Clostridium butyricum* and *Bacillus subtilis* supplementation. In contrast, Khoobani et al. [10] reported significant improvements in BWG and FCR with chicory powder and probiotic supplementation, although FI remained unaffected.

A different response pattern was observed for mortality, with prophylactic PBB resulting in the lowest mortality (4.17%) and numerically higher EPEF among the supplemented groups compared with MSP supplementation. The PC+PBB (Ther) group exhibited higher mortality (20.83%) and lower EPEF than the corresponding prophylactic group. The lower mortality observed in the PC+PBB (Prophy) group may reflect the potential effects of plant bioactive compounds. Previous studies have reported antioxidant and anti-inflammatory properties of bioactive compounds present in grape byproducts [23] however, these effects were not directly evaluated in the present study. Although EPEF differences were non-significant (p>0.05), the lower mortality in the prophylactic group suggests a biologically meaningful protective effect of early PBB administration, with greater protection against APEC than therapeutic intervention after clinical onset.

Likewise, Farouk et al. [24] demonstrated that, rosemary supplementation (0.5 g/kg) lowered mortality in *E. coli*-challenged broilers. In divergence, the EPEF was significantly (p<0.05) increased in 7-day-old Muscovy ducks over a duration of 70 days when they were provided with grape pomace at levels of 2.5%, 5%, and 7.5% [25]. Antioxidant, oxidative stress, histomorphological, and molecular parameters were not assessed, limiting conclusions regarding the underlying mechanisms.

Dietary incorporation of PBB under *E. coli* challenge conditions significantly reduced fecal *E. coli* counts compared with the PC group. Similar findings were reported by Fathi et al. [26], who observed reduced cecal *E. coli* counts in broilers supplemented with chicory processing wastewater. Likewise, inulin supplementation at 1% did not effect on fecal *E. coli* counts [27]. In contrary, Hayatolgheibi et al. [28] reported no significant effect of probiotics on caecal *E. coli* counts in dexamethasone-challenged birds. The reduction in *E. coli* may be attributed to chicory-derived inulin [29], a prebiotic substrate that promotes beneficial bacteria and short-chain fatty acid production, lowering intestinal pH and suppressing pathogenic *E. coli* proliferation [10].

Total *E. coli* enumeration was performed and did not differentiate the challenged APEC strain from the resident intestinal *E. coli*. Specific identification of the challenged APEC strain would require molecular methods (e.g., qPCR targeting *fimC*, *papC*, and *iucD*) or strain-specific markers, which were beyond the scope of the present study. Although total *E. coli* counts indicate bacterial burden, strain-specific quantification and assessment of bacterial dissemination to internal organs would provide stronger microbiological evidence.

Birds in the group PC+PBB and PC+PBB (Prophy) showed a mild abdominal air sac lesion score. Similar findings were reported by Thakur et al. [30] who observed mild air sacculitis in *E. coli*-challenged broilers receiving *Artemisia nilagirica* leaf extract. Likewise, Redweik et al. [31] reported reduced signs of air sacculitis in air sac–challenged white leghorn chicks supplemented with probiotics.

The therapeutic regimen was designed to simulate a practical field situation in which treatment is initiated after the appearance of clinical signs rather than immediately following pathogen exposure. Although APEC-associated colisepticemia can progress rapidly, commercial poultry operations often rely on flock-level clinical observations before initiating intervention through the drinking water. Therefore, the water-based therapeutic model reflects current field practices rather than an idealized experimental scenario. The comparatively lower efficacy observed with therapeutic administration, compared with prophylactic application, likely reflects the advanced stage of infection at the time treatment was initiated.

Numerical variations in hematological parameters reflected physiological responses to *E. coli* challenge and dietary treatments. The PC group showed increased TEC and reduced TLC compared with NC, whereas PBB and MSP supplementation had no consistent effects. The comparable H: L ratio among treatments indicated maintenance of physiological homeostasis. Similar findings were reported by Tayer et al. [32], who observed no significant changes in RBCs, WBCs, Hb, PCV, or lymphocyte percentage following grape leaf supplementation. Likewise, Hayatolgheibi et al. [28] found no significant effect of probiotic supplementation on eosinophil and lymphocyte counts in challenged birds. In contrast, Talaat and Elnaggar [25] reported improved hematological parameters in Muscovy ducks fed grape pomace.

The serum biochemical results were consistent with Huang et al. [33], who reported no significant effects of rosemary extract on ALT, ALB, and A: G ratio, and Khoobani et al. [10], who found no changes in TP and ALB following chicory supplementation. Conversely, Apaydın Yildirim et al. [34] observed increased ALT, AST, TP, and ALB in rosemary-supplemented broilers, while Talaat and Elnaggar [25] reported increased TP, GBL, and AST, with no effect on ALB and ALT, in grape pomace-fed ducks. Overall, prophylactic PBB supplementation may help maintain physiological stability and alleviate hepatic stress under challenge conditions.

No formal power analysis was performed during study planning. One limitation of the present study is that lesion scoring and hematological or serum biochemical analyses were conducted using one bird randomly selected from each replicate (n = 4 birds per treatment group), an approach consistent with previously reported experimental designs [13]. Although this approach ensured independent sampling across replicates, the relatively small sample size and potential biological variability among challenged birds may have limited the statistical power to detect subtle treatment effects. Therefore, these findings should be interpreted cautiously and confirmed in future studies with larger sample sizes to improve statistical power and robustness.

Collectively, these findings do not suggest that the phytogenic formulation should replace antibiotics as a standalone therapy in severe acute colibacillosis. Instead, they indicate that the phytogenic formulation may have value as part of an integrated health management strategy, while prophylactic use appears to offer greater benefits under challenged conditions.

## CONCLUSION

No significant differences were observed among groups for growth performance, EPEF, or cecal *E. coli* load. Prophylactic PBB supplementation resulted in lower mortality than the PC and MSP groups. For fecal *E. coli* load, both PC+PBB and PC+MSP groups showed significant reductions compared with PC, while no significant difference was observed between PBB and MSP. Overall, PBB and MSP showed comparable responses under challenged conditions; however, these findings should be interpreted cautiously and validated through further studies with larger sample sizes and targeted microbiological assessments.

## Figures and Tables

**Table 1:** Description of dietary treatments

Experimental groups	Treatment
Negative control (NC)	Basal diet+no APEC challenge+no gut acting antibacterial agents
Positive control (PC)	Basal diet+APEC challenge at 10 d of age (10^8^ CFU/bird)+no gut acting antibacterial agents
NC+PBB	Basal diet+no APEC challenge+PBB through feed from 1 to 42 d at dose of 100 g/100 kg of feed
PC+PBB	Basal diet+APEC challenge+PBB through feed from 1 to 42 d at dose of 100 g/100 kg of feed
PC+PBB (Prophy)	Basal diet+APEC challenge+PBB (100 g/100 kg of feed) from 1 to 42 d+additional PBB through drinking water (2 mL/L) as prophylactic dose on 7^th^ to 9^th^ day and 11^th^ to 17^th^ d for 12 h daily (06:00–18:00 h).
PC+PBB (Ther)	Basal diet+APEC challenge+PBB (100 g/100 kg of feed) from 1 to 42 d+additional PBB through drinking water (2 mL/L) as therapeutic dose for up to 10 consecutive days following the onset of clinical signs.
PC+MSP	Basal diet+APEC challenge at 10 d of age (10^8^ CFU/bird)+MSP at dose of 200 mg/kg (*Bacillus* subtilis-based) through feed from 1 to 42 d
PC+MSP (Ther)	Basal diet+APEC challenge+MSP (200 mg/kg of feed) from 1 to 42 d+additional MSP through drinking water (2 g/50 birds) for 6 h for 5 d under therapeutic conditions upon onset of clinical signs

APEC, Avian pathogenic *Escherichia coli*; PBB, phytogenic botanical blend; Prophy, prophylactic; Ther, therapeutic; MSP, multi strain probiotic; CFU, colony forming units.

**Table 2:** Composition and calculated nutrient composition of basal diet

Ingredients (%)	Starter	Grower	Finisher
Maize	58.24	63.27	65.67
Soybean meal Hi Pro	34.01	30.02	27.26
Rice bran oil	3.37	3.21	4.15
Limestone powder	1.41	1.03	0.88
Mono calcium phosphate	1.17	0.82	0.55
DL-methionine	0.40	0.36	0.32
L-lysine HCl	0.28	0.25	0.22
L-threonine	0.17	0.20	0.11
L-valine	0.05	0.04	0.03
L-arginine	0.02	0.02	0.01
Salt	0.27	0.20	0.20
Sodium-bicarbonate	0.20	0.20	0.20
Potassium carbonate	0.15	0.15	0.15
Choline chloride	0.12	0.10	0.10
Vitamin premix1)	0.05	0.05	0.05
Trace mineral premix2)	0.05	0.05	0.05
Diclazuril 0.5%	0.03	-	-
Salinomycin 5%	-	0.05	0.05
Phytase 10G	0.01	0.01	0.01
Nutrients	Starter	Grower	Finisher
AME (kcal/kg)	2,975	3,050	3,150
Dry matter (%)	88.86	89.25	89.05
Organic matter (%)	94.35	95.18	95.55
Ether extract (%)	6.25	5.85	6.82
Crude protein (%)	22.82	20.58	18.6
Crude fiber (%)	2.8	2.65	2.45
Calcium (%)	0.95	0.75	0.65
Total phosphorus (%)	0.65	0.56	0.49
Available phosphorus (%)	0.5	0.42	0.36
Phytate phosphorus (%)	0.26	0.26	0.25
SID amino acids			
Lysine (%)	1.32	1.18	1.08
Methionine (%)	0.7	0.64	0.59
Methionine+cystine (%)	1	0.92	0.86
Threonine (%)	0.88	0.85	0.72
Tryptophan (%)	0.24	0.21	0.2
Arginine (%)	1.4	1.27	1.17
Isoleucine (%)	0.88	0.8	0.75
Valine (%)	1	0.91	0.84
Leucine (%)	1.68	1.57	1.49
Histidine (%)	0.54	0.49	0.46
Phenylalanine (%)	1	0.92	0.86
Glycine (%)	0.58	0.5	0.45
Serine (%)	0.69	0.6	0.54
Sodium (%)	0.22	0.2	0.19
Chloride (%)	0.22	0.19	0.17
Potassium (%)	0.98	0.9	0.85
Choline (mg/kg)	1,700	1,642	1,579

1) The vitamin premix supplied the following per kg of diet: 50 mg vitamin E, 13,000 IU vitamin A (4.47 mg retinyl acetate), 4,000 IU vitamin D (0.1 mg), 15 mg pantothenic acid, 9 mg riboflavin, 5 mg pyridoxine, 3 mg thiamine, 3 mg menadione, 2 mg folic acid, 60 mg niacin, 0.15 mg biotin, and 0.02 mg vitamin B_12_.

2) The trace mineral premix supplied the following per kg diet: 30 mg zinc (as yeast proteinate), 15 mg iron (as yeast proteinate), 5 mg copper (as yeast proteinate), 30 mg manganese (as yeast proteinate), 0.30 mg selenium (as yeast proteinate), 0.50 mg chromium (as yeast proteinate), and 2 mg iodine (as potassium iodide).

AME, apparent metabolizable energy; SID, standardized ileal digestible.

**Table 3:** Effect of PBB and MSP both powder and liquid based formulations on live body weight (g) of broiler chickens under APEC challenge

Days	NC	PC	PBB				MSP		SEM	p-values
	
			NC	PC	PC (Prophy)	PC (Ther)	PC	PC (Ther)		
0^th^	45.34	45.50	45.23	45.69	45.63	45.21	45.21	45.73	0.08	0.14
10^th^	293.04	295.36	293.81	297.98	299.33	303.21	297.13	295.83	1.16	0.42
24^th^	1,148.46	1,068.35	1,155.65	1,127.69	1,139.60	1,092.29	1,106.85	1,169.40	12.16	0.54
35^th^	2,213.18	2,200.08	2,343.32	2,344.22	2,285.93	2,245.33	2,323.94	2,231.98	20.75	0.33
42^nd^	2,925.52	2,921.99	3,123.39	3,108.11	3,062.71	2,965.14	3,107.58	3,013.35	29.57	0.68

PBB, phytogenic botanical blend; MSP, multi strain probiotic; APEC, Avian pathogenic *Escherichia coli*; NC, negative control; PC, positive control; Prophy, prophylactic; Ther, therapeutic, SEM, standard error of the mean.

**Table 4:** Effect of PBB and MSP both powder and liquid based formulations on body weight gain (g) of broiler chickens under APEC challenge

Days	NC	PC	PBB				MSP		SEM	p-values
	
			NC	PC	PC (Prophy)	PC (Ther)	PC	PC (Ther)		
0–10	247.71	249.86	248.59	252.29	253.71	258.00	251.92	250.11	1.16	0.43
11–24	855.42	773.00	861.83	829.71	840.27	789.08	809.73	873.56	12.67	0.49
25–35	1,064.72	1,131.72	1,187.67	1,216.54	1,146.33	1,153.03	1,217.09	1,062.58	21.37	0.31
36–42	712.34	721.92	780.07	763.89	776.78	719.82	783.63	781.37	11.06	0.96
0–24	1,103.12	1,022.85	1,110.42	1,082.00	1,093.98	1,047.08	1,061.64	1,123.67	12.14	0.54
0–35	2,167.85	2,154.58	2,298.09	2,298.54	2,240.31	2,200.12	2,278.73	2,186.25	20.76	0.33
0–42	2,880.19	2,876.49	3,078.16	3,062.42	3,017.08	2,919.93	3,062.37	2,967.62	29.57	0.68

PBB, phytogenic botanical blend; MSP, multi strain probiotic; APEC, Avian pathogenic *Escherichia coli*; NC, negative control; PC, positive control; Prophy, prophylactic; Ther, therapeutic, SEM, standard error of the mean.

**Table 5:** Effect of PBB and MSP both powder and liquid based formulations on feed intake (g) of broiler chickens under APEC challenged

Days	NC	PC	PBB				MSP		SEM	p-values
	
			NC	PC	PC (Prophy)	PC (Ther)	PC	PC (Ther)		
0–10	257.27	261.08	261.85	267.06	259.65	265.83	260.85	258.75	1.19	0.32
11–24	1,200.63	1,258.27	1,203.17	1,269.09	1,181.46	1,287.88	1,251.93	1,287.29	14.72	0.24
25–35	1,684.48	1,669.82	1,786.30	1,774.63	1,739.33	1,689.34	1,735.33	1,727.18	14.94	0.74
36–42	1,441.45	1,512.81	1,436.02	1,402.42	1,368.70	1,353.55	1,456.48	1,438.95	17.96	0.63
0–24	1,457.90	1,519.36	1,465.02	1,536.15	1,441.11	1,553.72	1,512.78	1,546.04	15.35	0.21
0–35	3,142.37	3,189.18	3,251.32	3,310.79	3,180.44	3,243.05	3,248.12	3,273.22	19.43	0.85
0–42	4,583.83	4,701.99	4,687.33	4,713.21	4,549.13	4,596.60	4,704.60	4,712.17	23.92	0.95

PBB, phytogenic botanical blend; MSP, multi-strain probiotic; APEC, Avian pathogenic *Escherichia coli*; NC, negative control; PC, positive control; Prophy, prophylactic; Ther, therapeutic, SEM, standard error of the mean.

**Table 6:** Effect of PBB and MSP both powder and liquid based formulations on FCR of broiler chickens under APEC challenged

Days	NC	PC	PBB				MSP		SEM	p-values
	
			NC	PC	PC (Prophy)	PC (Ther)	PC	PC (Ther)		
0–10	1.039	1.045	1.053	1.059	1.023	1.030	1.035	1.035	0.00	0.41
11–24	1.404	1.628	1.396	1.530	1.406	1.632	1.546	1.474	0.03	0.14
25–35	1.582	1.475	1.504	1.459	1.573	1.465	1.426	1.625	0.03	0.10
36–42	2.024	2.096	1.841	1.836	1.762	1.880	1.859	1.842	0.04	0.78
0–24	1.322	1.485	1.319	1.420	1.317	1.484	1.425	1.376	0.03	0.13
0–35	1.450	1.480	1.415	1.440	1.420	1.474	1.425	1.497	0.01	0.12
0–42	1.592	1.635	1.523	1.539	1.508	1.574	1.536	1.588	0.02	0.30

PBB, phytogenic botanical blend; MSP, multi-strain probiotic; FCR, feed conversion ratio; APEC, Avian pathogenic *Escherichia coli*; NC, negative control; PC, positive control; Prophy, prophylactic; Ther, therapeutic, SEM, standard error of the mean.

**Table 7:** Effect of PBB and MSP both powder and liquid based formulations on mortality % and EPEF of broiler chickens under APEC challenged

	NC	PC	PBB				MSP		SEM	p-values
	
			NC	PC	PC (Prophy)	PC (Ther)	PC	PC (Ther)		
0–10	0.00	0.00	0.00	0.00	0.00	0.00	0.00	0.00	-	-
11–24	0.00	14.58	0.00	10.42	4.17	14.58	10.42	8.33	2.07	0.03
25–35	0.00	0.00	0.00	0.00	0.00	2.44	0.00	2.27	0.39	0.52
36–42	2.08	2.44	2.08	2.33	0.00	5.00	4.65	2.33	0.56	0.94
0–24	0.00	14.58	0.00	10.42	4.17	14.58	10.42	8.33	2.07	0.03
0–35	0.00	14.58	0.00	10.42	4.17	16.67	10.42	10.42	2.23	0.02
0–42	2.08	16.67	2.08	12.50	4.17	20.83	14.58	12.50	2.50	0.02
EPEF	429.92	359.10	478.63	420.39	465.96	357.29	411.20	396.32	15.65	0.06

PBB, phytogenic botanical blend; MSP, multi strain probiotic; EPEF, European production efficiency factor; APEC, Avian pathogenic *Escherichia coli*; NC, negative control; PC, positive control; Prophy, prophylactic; Ther, therapeutic, SEM, standard error of the mean.

**Table 8:** Effect of PBB and MSP both powder and liquid based formulations on broiler chickens under APEC challenge for *Escherichia coli* (log_10_ CFU/g) enumeration

Sample	Day (d)	NC	PC	PBB				MSP		SEM	p-values
	
				NC	PC	PC (Prophy)	PC (Ther)	PC	PC (Ther)		
Cecal content	14	5.93	6.57	6.08	5.59	6.19	6.39	6.18	6.54	0.12	0.055
Fecal content	9	6.86	6.58	6.61	6.15	6.26	6.71	7.03	5.64	0.16	0.053
	16	7.02^b^	7.31^b^	7.14^b^	6.43^a^	7.15^b^	6.58^a^	6.49^a^	7.06^b^	0.12	0.001

a,b Means with dissimilar superscripts in a row varied significantly (p<0.05).

PBB, phytogenic botanical blend; MSP, multi strain probiotic; APEC, Avian pathogenic *E. coli*; log_10_ CFU/g, logarithm (base 10) of colony-forming units per gram; NC, negative control; PC, positive control; Prophy, prophylactic; Ther, therapeutic; SEM, standard error of the mean.

**Table 9:** Effect of PBB and MSP both powder and liquid based formulations on lesion score of visceral organs (n = 4)

Parameters	NC	PC	PBB				MSP	
	
			NC	PC	PC (Prophy)	PC (Ther)	PC	PC (Ther)
Body emaciation	0 (4)	0 (4)	0 (4)	0 (4)	0 (4)	0 (4)	0 (4)	0 (4)
Trachea	1 (1), 0 (3)	0 (4)	0 (4)	0 (4)	0 (4)	0 (4)	0 (4)	0 (4)
Lungs	0 (4)	0 (4)	0 (4)	0 (4)	0 (4)	0 (4)	0 (4)	0 (4)
Thoracic air sac	0 (4)	1 (1), 0 (3)	0 (4)	0 (4)	0 (4)	0 (4)	0 (4)	0 (4)
Abdominal air sac	1 (1), 0 (3)	1 (1), 2 (1), 0 (2)	0 (4)	1 (1), 1 (1), 0 (2)	1 (1), 1 (1), 0 (2)	0 (4)	1 (1), 0 (3)	1 (1), 1 (1), 0 (2)
Liver	0 (4)	0 (4)	0 (4)	0 (4)	0 (4)	0 (4)	0 (4)	0 (4)

Gross lesion scores were assessed in one bird randomly selected from each of the four replicates (n = 4 birds per treatment group).

Lesion score: 0 (normal), 1 (mild), 2 (moderate), 3 (severe).

Values in brackets indicate the number of birds with the respective lesion score.

PBB, phytogenic botanical blend; MSP, multi strain probiotic; NC, negative control; PC, positive control; Prophy, prophylactic; Ther, therapeutic.

**Table 10:** Effect of PBB and MSP both powder and liquid based formulations on blood parameters of broiler chickens under APEC challenge

Parameters	NC	PC	PBB				MSP		SEM	p-values
	
			NC	PC	PC (Prophy)	PC (Ther)	PC	PC (Ther)		
TEC (×10^6^/μL)	1.95	2.88	2.77	3.42	2.31	2.27	2.03	2.03	3	0.18
TLC (×10^3^/μL)	10.43	7.50	7.70	5.88	7.79	8.60	6.75	6.75	9.91	0.54
Heterophil	16.00	14.75	11.50	15.25	14.75	15.75	14	14	13.75	0.51
Lymphocyte	75.75	78.25	78	73	71.75	76.50	74.25	74.25	77	0.83
H:L	0.29	0.19	0.15	0.21	0.21	0.21	0.19	0.19	0.18	0.01
Monocyte	5.75^ab^	4.75^a^	7.50abc	8.75^bc^	10.25^c^	5.25^a^	9.50^c^	9.50^c^	7.50abc	0.004
Eosinophil	2.50	2.25	3	3	2.75	2.50	2.25	2.25	1.75	0.15
AST (U/L)	143.25^a^	170.75^c^	147.50^ab^	169.25^c^	152^b^	189.50^d^	168.25^c^	201.25^e^	7.15	<0.0001
ALT (U/L)	9.25	11.03	12.78	10.30	10.98	8.73	9.70	10.33	0.44	0.071
ALP (U/L)	42.50^c^	39.25^c^	28^b^	64.25^e^	48.50^d^	22.25^a^	30.50^b^	31.75^b^	4.75	<0.0001
Total bilirubin (g/L)	4.3^ab^	4.6abc	4.08^a^	5.2^c^	4.4^ab^	5.3^c^	5.1^bc^	4.4^ab^	0.02	0.011
TP (g/L)	24.5^a^	24.8^a^	23.2^a^	25.5^a^	24.0^a^	25.5^a^	23.3^a^	28.5^b^	0.06	0.006
Albumin (g/L)	16.5	15.5	15.4	14.1	16.1	14.9	16.3	15.7	0.03	0.091

a–e with dissimilar superscripts in a row varied significantly (p<0.05).

PBB, phytogenic botanical blend; MSP, multi strain probiotic; APEC, Avian pathogenic *Escherichia coli*; NC, negative control; PC, positive control; Prophy, prophylactic; Ther, therapeutic; SEM, standard error of the mean; TEC, total erythrocyte counts; TLC, total leukocyte counts; H:L, heterophil to lymphocyte ratio; AST, aspartate amino transferase; ALT, alanine amino transferase; ALP, Alkaline phosphatase; TP, total protein.

## Data Availability

Upon reasonable request, the datasets of this study can be available from the corresponding author.
